# Superparamagnetic Iron Oxide Nanoparticles Labeling of Bone Marrow Stromal (Mesenchymal) Cells Does Not Affect Their “Stemness”

**DOI:** 10.1371/journal.pone.0011462

**Published:** 2010-07-07

**Authors:** Arun Balakumaran, Edyta Pawelczyk, Jiaqiang Ren, Brian Sworder, Aneeka Chaudhry, Marianna Sabatino, David Stroncek, Joseph A. Frank, Pamela G. Robey

**Affiliations:** 1 Craniofacial and Skeletal Diseases Branch, National Institute of Dental and Craniofacial Research, National Institutes of Health, Bethesda, Maryland, United States of America; 2 Radiology and Imaging Sciences, Clinical Center, National Institutes of Health, Bethesda, Maryland, United States of America; 3 Department of Transfusion Medicine, Clinical Center, National Institutes of Health, Bethesda, Maryland, United States of America; 4 National Institute of Biomedical Imaging and Bioengineering, National Institutes of Health, Bethesda, Maryland, United States of America; Universität Heidelberg, Germany

## Abstract

Superparamagnetic iron oxide nanoparticles (SPION) are increasingly used to label human bone marrow stromal cells (BMSCs, also called “mesenchymal stem cells”) to monitor their fate by *in vivo* MRI, and by histology after Prussian blue (PB) staining. SPION-labeling appears to be safe as assessed by *in vitro* differentiation of BMSCs, however, we chose to resolve the question of the effect of labeling on maintaining the “stemness” of cells within the BMSC population *in vivo*. Assays performed include colony forming efficiency, CD146 expression, gene expression profiling, and the “gold standard” of evaluating bone and myelosupportive stroma formation *in vivo* in immuncompromised recipients. SPION-labeling did not alter these assays. Comparable abundant bone with adjoining host hematopoietic cells were seen in cohorts of mice that were implanted with SPION-labeled or unlabeled BMSCs. PB+ adipocytes were noted, demonstrating their donor origin, as well as PB+ pericytes, indicative of self-renewal of the stem cell in the BMSC population. This study confirms that SPION labeling does not alter the differentiation potential of the subset of stem cells within BMSCs.

## Introduction

Two FDA-approved agents, ferumoxides (Fe), a suspension of superparamagnetic iron oxide nanoparticles (SPION), and protamine sulfate (Pro), a drug used to reverse heparin anticoagulation, have been combined (FePro) and used to magnetically label cells, including stem and progenitor cells [1], [2]. Labeling stem cells with SPION allows for the non-invasive monitoring by MRI in both animal and human trials [3]–[7]. SPION-labeled cells can also be detected using Prussian blue (PB) stain to correlate histology to MRI. Such non-invasive and sensitive imaging techniques in the future will be valuable for optimizing cell therapy, especially stem or progenitor cells, by tracking labeled cells after infusion.

Multiple reports have documented safety of this labeling technique, such as no short- or long-term toxic effects, no production of reactive oxygen species, no modification in the viability and proliferation and cellular function or phenotype of SPION-labeled compared to unlabeled BMSCs [8], [9]. However, in order for labeled stem cell trial therapies to be effective, this technique must not alter the “stemness” (their ability to regenerate a tissue and to self-renew). We have shown that FePro labeling does not alter the *in vitro* differentiation capacity of CD34 positive hematopoietic stem cells (HSCs) or bone marrow stromal cells (BMSC) to osteogenic or adipogenic cells [9], [10]. SPION labeling was also shown not to inhibit cartilaginous differentation, [10] but another group found a decrease in cartilaginous differentation when higher concentrations of the SPION were used [11]. None of the studies have performed an *in vivo* differentiation assay.

The current assay to establish stem cell function of differentiation and self-renewal, is exemplified by the capacity of a single prospectively isolated HSC to reconstitute, serially and long term, multilineage hematopoiesis in lethally irradiated recipient mice. Progress has been made towards developing an equivalent “gold standard” assay for human BMSCs. Multipotency of BMSCs is commonly assessed by *in vitro* differentiation assays. However, these assays correlate poorly with results of *in vivo* differentiation assays, even when conducted in parallel on the same cell strain [12], [13]. Furthermore, multipotency (a property of a single cell) cannot be determined based on assays conducted on non-clonal cell strains in culture. *In vitro* generation of alizarin red deposits (osteogenesis), oil red O-stainable cells (adipogenesis), and alcian blue-stainable matrix (chondrogenesis) in parallel cultures of non-clonal strains of BMSCs, or any strain of cells, does not predict multipotency of a single cell [12]. The ability of BMSCs mixed with appropriate carrier to make donor-derived bone and hematopoietic-supporting stroma, frequently called an “ossicle” in a immunocompromised mouse, establishes the stem cell potential of the tested BMSC population [14]. Colony forming efficiency of the BMSCs and their CD146 expression are an indirect measure of the stem cell content of a BMSC population even though they do not replace the in vivo assay [14]. In this study, we examined whether FePro labeling affects the “stemness” of BMSCs, as defined by their ability to differentiate and self-renew, by determining the ability of labeled BMSCs to make ossicles, colony forming efficiency, CD146 expression in addition to gene profiling of labeled versus unlabeled cells.

## Results

FePro labeling of BMSCs resulted in approximately 100% of cells being labeled after counting Prussian blue positive cells.

### Equivalent Colony forming efficiency after FePro labeling

**Figure 1:**
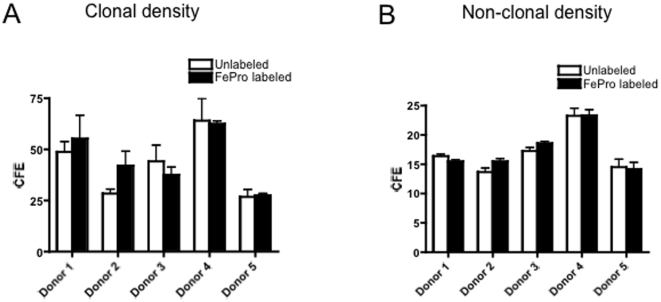
Colony forming efficiency in FePro-labeled BMSCs. Secondary colony forming efficiency of BMSCs plated at clonal density (A) or high density (B) from 5 donors. Data are represented as mean +/− S.D. of colony forming units for each donor done in triplicates. Note the lack of a statistically different change in number of colonies in SPION-labeled BMSCs (solid colored bars), Student t test, p>0.5. A similar lack of a statistically different change in the number of colonies were found when the secondary colony forming efficiency experiments from 5 donors were repeated independently by two other scientists.

FePro labeling did not lead to an alteration in the colony forming ability of BMSCs in assays with varying plating density and when conducted independently by at least two of the authors. The number of colonies was higher for both FePro labeled and unlabeled BMSCs when plated at clonal density compared to high-density plating (see Figure 1A and 1B).

### Lack of difference in CD146 expression after SPION labeling

**Figure 2:**
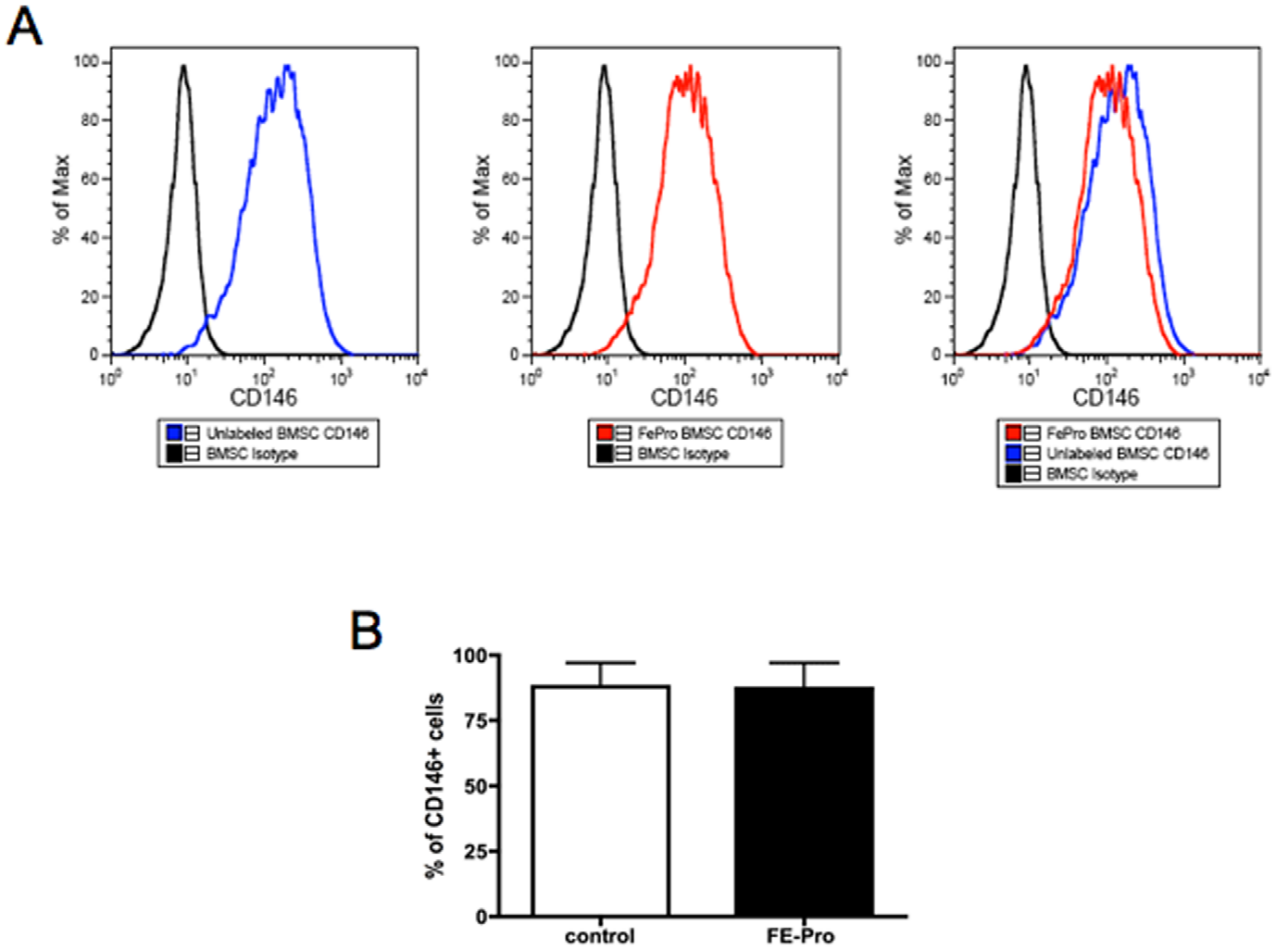
CD 146 expression in FePro-labeled BMSCs. (A) Representative flow cytometry histogram with overlay of the two groups showing no difference in CD146 expression after SPION labeling of BMSCs. (B) Bar graph showing mean CD146 expression in FePro labeled and unlabeled BMSC. Note the lack of statistically significant difference in CD146 expression after SPION labeling (solid colored bars), Student t test, p>0.5. Data shown as mean +/− S.D. of CD 146 expression in 5 donors.

FePro labeling did not alter the CD146 expression in any of the 5 donors tested (see Figure 2). A large percentage of the BMSCs both in labeled and unlabeled cells in different donors were CD 146 positive cells.

### Concordant gene expression profiles

**Figure 3:**
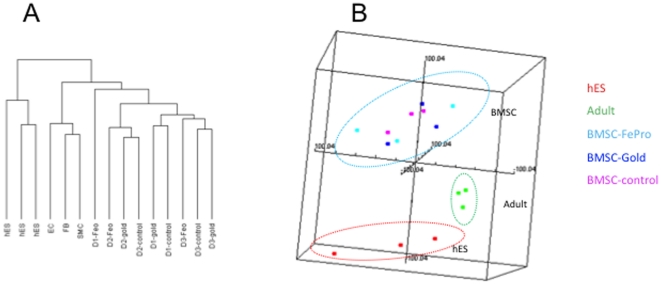
Global gene expression and multidimensional scaling analysis of FePro labeled BMSCs. BMSC samples from 3 donors (FePro-labeled, gold nanoparticle-labeled and unlabeled control) and control cells (3 samples from human embryonic stem cells and 3 samples of adult cells) were analyzed by an oligonucleotide microarray. The multidimensional scaling plot similarly grouped the hES cells together, the adult cells other than BMSCs together in another group, and all the BMSC samples into a third group. The BMSCs did not cluster according to the type of labeling method. hES- human embryonic stem cell; adult indicated the adult cells: Fb-fibroblasts, EC endothelial cells, SMC-smooth muscle cells; BMSC-FePro: bone marrow stromal cellslabeled with FePro; BMSC-Gold: bone marrow stromal cells labeled with gold nanoparticle; BMSC-control: unlabeled BMSC control; D1: donor 1; D2-donor 2; D3 donor 3.

Recently, gene expression profiling has been used to determine stem cells and to test the potency of cellular therapies [15]. We used global transcriptional profiling to evaluate the potential effect of FePro labeling on BMSCs. As a control to FePro -labeled cells, we labeled BMSCs with gold nanoparticles. SPIONs, once internalized by the cell undergo progressive degradation [9], while gold nanoparticles are inert. Among more than 36,000 probes in the array, only those genes that were expressed by greater than 80% of BMSCs and whose fold change more than 1.5 were selected for analysis. The resulting 8,506 genes were analyzed by unsupervised hierarchical clustering and multidimensional scaling analysis. The 15 samples were grouped into 3 clusters: one with all 3 hES cells, one with the 3 adult cells: fibroblasts, smooth muscle cells and endothelial cells, and another with all BMSCs. Among the BMSC samples, there was no segregation of samples according to the labeling method. Similarly, a multidimensional scaling analysis classified the 3 hES samples into one group, the 3 adult cells into a second group, and the 12 BMSC samples into the third group (Figure 3). The BMSC samples did not cluster according to the labeling method (Figure 3B). Only 72 differentially expressed genes were identified among the three groups. Among the genes that were changed (at least 2 fold, F-test, *P*<0.01) in FePro-labeled BMSCs compared to unlabeled BMSCs or gold nanoparticle labeled BMSCs were gene families related to ion binding, ion or vesicle transport, cytoskeleton related genes or genes involved in the signal transduction pathways associated with cytoskeletal changes. With regard to genes involved in iron metabolism, ferritin and iron storage proteins were upregulated in FePro-labeled BMSCs and transferrin receptor was not changed.

### Comparable ability of labeled BMSCs to form a bone/marrow organ *in vivo*

**Figure 4:**
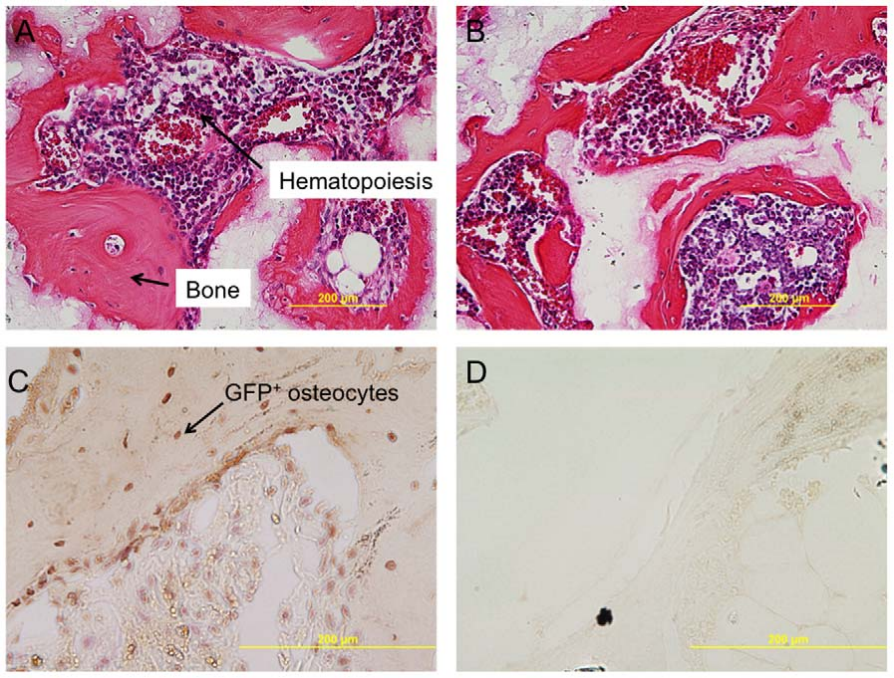
Immunohistochemical staining of ossicles derived from FePro or GFP labeled and unlabeled BMSCs. A representative ossicle derived from unlabeled (A) and FePro labeled (B) BMSCs at 8 weeks, stained with H & E showing comparable abundant bone formation and abundant hematopoiesis. Immunohistochemistry staining for GFP of a representative ossicle derived from BMSCs labeled with both FePro and lentivirus carrying GFP (C) and control unlabeled BMSCs (D).

**Figure 5:**
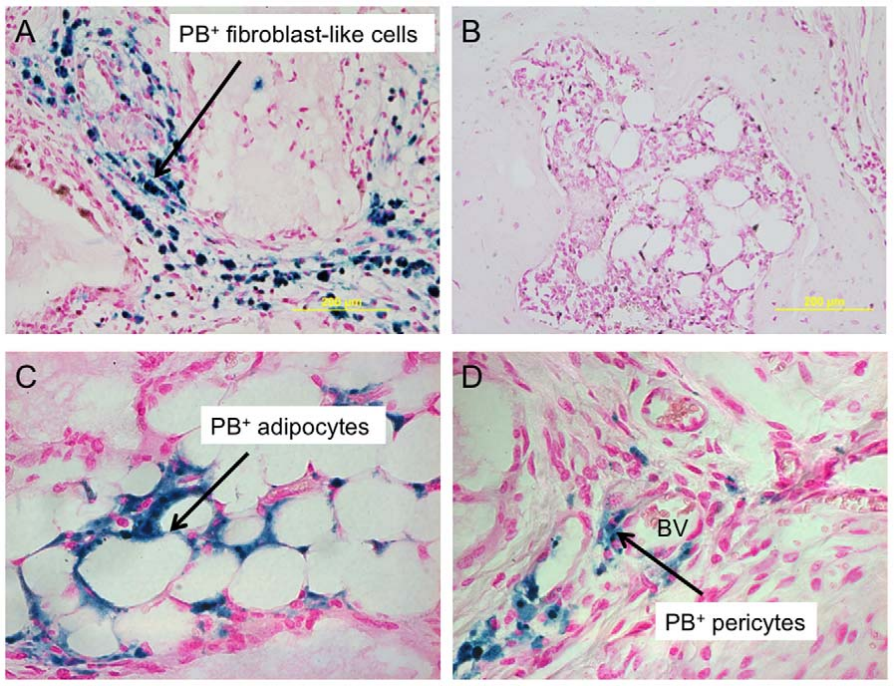
Prussian blue staining of ossicles derived from FePro or unlabeled BMSCs. Prussian blue (PB) staining of a representative ossicle derived from BMSCs labeled with FePro (A) and control unlabeled BMSCs (B). PB staining of a representative ossicle derived from BMSCs labeled with FePro showing PB^+^ adipocytes (C). PB staining of a representative ossicle derived from labeled BMSCs showing PB^+^ pericytes (D).

After 8 weeks, abundant bone formation supporting hematopoiesis was found in the transplants of FePro-labeled BMSCs (see Figure 4A), GFP-labeled BMSCs (see Figure 4A), both FePro- and GFP-labeled, or non-labeled BMSCs. The bone formation scores were similar between the four groups for each of the donors. In transplants of FePro- or GFP- labeled cells or both, PB or GFP positive cells were readily detectable (see Figure 4C and figure 5A). They appeared as stromal/fibroblast-like cells within areas of fibrous tissue and over carrier surfaces.

## Discussion

The closest approximation to estimating the frequency of stem cells is derived from the primary colony forming efficiency (CFE) assay, which enumerates the number of Colony Forming Units-Fibroblast, approximately 1 in 5 of which are stem cells [16]. Since the only way of establishing ex vivo expanded BMSCs is after adherence, we used CFE assays on passaged cells at both clonal and high densities. The increased CFE when cells are plated at a clonal density is intriguing and further studies are needed to prove that the colonies formed at clonal density are also an indication that some of these are stem cells. Although it is unclear if CFE assays performed on passaged BMSCs are an estimate for the frequency of stem cells, we nevertheless, did not see any significant differences after FePro labeling.

In human bone marrow, CD146 positivity marks adventitial reticular cells, [14] a stromal cell type residing in a subendothelial position over the abluminal surface of BM sinusoids [17]. CD146 appears to be a marker for the in situ counterpart of the primary colony forming unit fibroblast, [14] and thus, possibly an indirect estimate of the stem cells in BMSCs. No differences in CD146 positivity were observed with labeling.

The results of gene profiling after unsupervised hierarchical clustering and multidimensional scaling analysis suggested that there are only minor differences in gene expression profiles between SPION-labeled, gold nanoparticles labeled and unlabeled control BMSCs [18]. No substantial change in FePro-labeled BMSCs was observed on any of the genes thought to be critical for “stemness” of embryonic and adult stem cells such WNT pathway genes, when compared to unlabeled BMSCs. The changes in genes involved in iron metabolism are consistent with our previously published study [9].

FePro-labeling of BMSCs does not affect their ability to differentiate into bone, marrow adipocytes, fibroblasts and hematopoietic supporting stroma *in vivo*.

**Figure 6:**
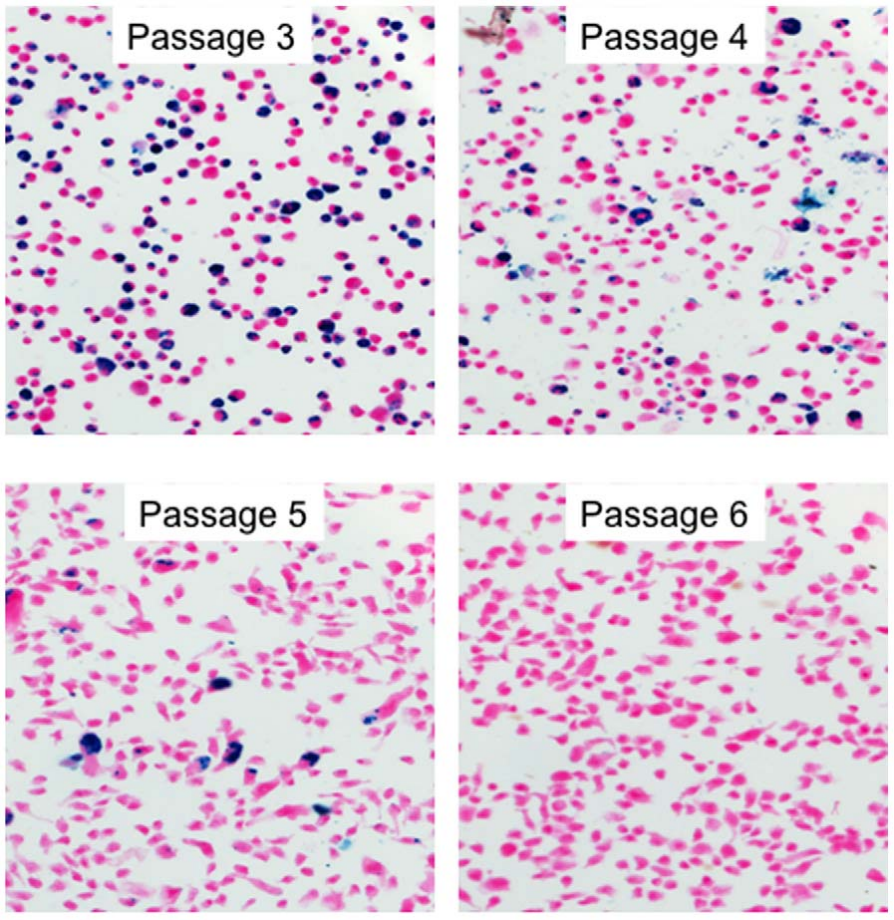
Dilution of FePro in cultured FePro-labeled and unlabeled BMSCs. The microphotographs show PB staining of BMSCs cultured *in vitro* at passages 3, 4, 5 and 6 after BMSCs at passage 2 were labeled with FePro.

The donor (human) origin of osteocytes and myelosupportive stroma have been determined by various methods, whereas the donor origin of marrow adipocytes has long been suspected and even assumed, but never proven owing to their large cytoplasmic to nuclear ratio, making detection with nuclear markers difficult. Unlike nuclear markers, labeled SPION, internalized by macropinocytosis, accumulate within endosome/lysosome compartment. Ossicles created with SPION-labeled cells show several Prussian blue positive adipocytes (see Figure 5C), strongly suggesting their origin from human BMSCs, although electron microscopy would be needed to be definitive. Prussian blue negative, GFP^+^ osteocytes (see Figure 4C) are probably the result of dilution of SPION label during the proliferation and subsequent differentiation of BMSCs. Labeled SPION are internalized by macropinocytosis, which accumulate within endosome/lysosome compartment. The label is diluted after a few passages of BMSCs (see figure 6). The intense proliferation of BMSCs necessary for bone formation *in vivo* possibly resulted in dilution of SPION label in the osteocytes.

The other distinct property of a stem cell is its capacity to self renew and the only system for which stem cell self-renewal is considered to be solidly proven is the hematopoietic system [19]–[21]. Evidence for self-renewal of the subset of multipotent BMSCs has only very recently started to emerge and is thought to be related to CD146 positivity [14]. It is beyond the scope of our objective to study self-renewal by serial passage of stroma from the “ossicles.” However, there is no data indicating that SPION labeling will alter the self-renewal capacity of BMSCs as PB+ pericytes were seen in the ossicles (see figure 5D) and CD146 expression is not different between FePro-labeled and unlabeled BMSCs (Figure 2). Ex vivo expanded labeled BMSCs reformed myelosupportive stroma.

In conclusion, our study shows that FePro-labeling of BMSCs does not affect their “stemness” in any of the fours assays that were utilized.

## Methods

### Harvest and expansion of human bone marrow stromal cells (BMSCs)

Briefly, bone marrow biopsies were obtained from volunteers after obtaining written informed consents under IRB of the National Institute of Dental and Craniofacial Institute approved procedures, and processed as described previously [12]. Fragments of trabecular bone and marrow were scraped gently with a steel blade into cold modified Minimum Essential Medium [alpha] MEM, (Life Technologies, Grand Island, NY). Released cells were pipeted, passed through 16- and 20-gauge needles and filtered through a 70-µm pore size nylon cell strainer (Becton Dickinson, Franklin Lakes, NJ). Single cell suspensions were plated at 1.0×10^7^ nucleated cells per 75-cm^2^ flask (Becton Dickinson, Lincoln Park, NJ). Cells were incubated at 37°C in a 95% air/5% CO_2_ atmosphere in growth medium containing [alpha]-MEM, 2 mM l-glutamine, 100 U/ml penicillin, 100 µg/ml streptomycin sulfate (Invitrogen, CA), and lot selected 20% fetal bovine serum. Medium was replaced on day 6 or 7. The cultures were passaged on day 12 with two consecutive applications of 1x trypsin-EDTA (Life Technologies, Gaithersburg, MD) for 5–10 min each at room temperature. Subsequent passages were performed at 4 to 7 day intervals. BMSCs were used at passages 3 or 4 for this study.

### Labeling of BMSC with Ferumoxides/Protamine Sulfate

Ferumoxides (Fe, Feridex IV, Berlex Laboratories, Wayne, NJ) are dextran coated SPIO nanoparticles approximately 120–150 nm in size and are provided at a total iron content of 11.2 mg/ml. Protamine sulfate (Pro, American Pharmaceuticals Partner, Schaumburg, IL), supplied at 10 mg/ml, was prepared as a fresh stock solution of 1 mg/mL in sterile distilled water immediately before labeling. Ferumoxides at a concentration of 100 µg/ml were put into a 50 ml conical tube containing serum-free RPMI 1640 (Biosource, Camarillo, CA) with 25 mM HEPES, MEM nonessential amino acids, sodium pyruvate, and L-glutamine. Protamine sulfate was added to the solution at 6 µg/ml and mixed for 2 minutes with intermittent hand shaking. Culture medium was aspirated from the flasks containing BMSCs and replaced with media containing FePro complexes. After 2 hours of incubation at 37°C, an equal amount of complete medium was added for a final concentration of Fe to Pro, 50 µg/ml to 3 µg/mL, respectively. Cells were incubated overnight (∼16 hours), and washed three times with sterile PBS containing 10 U/mL heparin sulfate (American Pharmaceuticals Partner, Schaumburg, IL). Complete medium was added to each flask and labeled cells were kept in culture for 2 days to ensure all FePro complexes were endocytosed.

### Labeling of BMSC with Gold nanoparticles

BMSCs were labeled in serum-free RPMI medium containing 2.25×10^7^ gold nanoparticles/mL (British Biocell International, Wales, U.K, particle size-250 nm) and 10 ng/mL protamine sulfate at 37C. After four hours, an equal volume of complete medium was added, and the cells were incubated overnight. The next day, the cells were washed three times with heparinized HBSS (10 U/mL) and returned to the incubator in normal medium. Gold nanoparticle labeling was performed for *in vitro* studies to evaluate alterations in gene expression as a result of cell labeling.

### Prussian Blue Staining and FePro Labeling Efficiency

To visualize the iron within FePro labeled cells, Prussian blue (PB) staining was performed. After 2 days post-labeling, BMSCs were trypsinized and transferred to cytospin slides. Cells were fixed with 4% glutaraldehyde, washed, and incubated for 30 minutes with 2% potassium ferric-ferrocyanide (Perl's reagent for staining, Sigma, St. Louis, MO) in 3.7% hydrochloric acid. Cells were washed again, counterstained with nuclear fast red and evaluated for iron staining using light microscopy (Axioplan Imaging II; Zeiss, Oberkochen, Germany) at 40×/0.75 objective lens and Axiovision 4.4 software (Zeiss, Oberkochen, Germany). FePro labeling efficiency was determined by manual counting of PB stained and unstained cells at 100× magnification using a 100×/1.30 oil immersion objective lens. The percentage of labeled cells was determined from the average of 5 high-powered fields. BMSCs labeled with gold nanoparticles were evaluated by generating optical differential interference contrast images (DIC) using Olympus BX-UCB microscope attached to a DP70 camera (Olympus, Center Valley, PA) at ×40/0.75 objective lens and MicroSuite™ Biological Suite software (Olympus, Center Valley, PA). The gold nanoparticle labeling efficiency was determined as described for FePro labeling.

### BMSCs transduction with lentivirus

BMSCs were transduced with lentivirus encoding for copeod green fluorescent protein (copGFP, pSIH1-siLuc-copGFP lentivirus, System Biosciences, Mountain View, CA) by replacing the medium with fresh medium containing viral particles and incubating overnight at 37°C in a 95% air per 5% CO_2_ atmosphere. After overnight incubation, media was replaced and cells incubated for 48 hours. The transfection efficiency was evaluated by flow cytometry and cells expressing the GFP transgene were sorted using the MoFlow cell sorter (Dako Cytomation, Fort Collins, CO).

### Colony Forming Efficiency

BMSCs at passage 2 or 3 were plated at concentrations of 41 (clonal density) or 500 (non-clonal density) nucleated cells in 25 cm^2^ flasks along with 6 mls of growth medium in order to determine secondary colony forming efficiency (CFE). The colony forming efficiency assay was performed by at least 2 of the authors independently for each of the donor BMSCs. After incubation for 10–14 days without medium change, cultures were washed with HBSS, fixed with 100% methanol and stained with an aqueous solution of saturated methyl violet. Using a dissecting microscope, colonies with greater than 50 cells are counted, and the CFE is determined per nucleated cells plated.

### Assay for bone formation and hematopoietic support *in vivo*

BMSCs of 3rd or 4th passages from 5 donors were used for the *in vivo* transplantation assay. The transplantation technique was performed as described in detail elsewhere [22]. Briefly, trypsinized and pelleted BMSCs were resuspended in 1 ml of standard medium. The cell suspensions were mixed with 40 mg of hydroxyapatite/tricalcium phosphate (HA/TCP) ceramic powder (particle size 0.5–1.0 mm, generously provided by Zimmer, Inc., Warsaw, IN), and the mixtures were incubated at 37°C for 90 min with slow rotation (25 rpm) prior to implantation.

Eight- to 15-week-old immunodeficient female beige mice (bg-nu/nu-xid, Charles River Laboratories, Raleigh, NC, or Harlan Sprague Dawley, Indianapolis, IN) were used as transplant recipients. All research involving animals were conducted according to the National Institutes of Health Animal Care and Use Guidelines. Animal experiments were performed according to a protocol approved by National Institute of Dental and Craniofacial Research's Animal Care and User Committee (ACUC) of National Institutes of Health. Animals were maintained under *ad libitum* diet supplied by Harlan Laboratories. Every effort was made to minimize the number of animals used and their suffering. Procedures were performed in accordance to specifications of an approved small animal protocol under anesthesia achieved with isoflurane in an induction chamber. Mid-longitudinal skin incisions of 1 cm length was made on the dorsal surface of each mouse, and up to four subcutaneous pockets were formed by blunt dissection. A single transplant was placed into each pocket. The incisions were closed with surgical staples.

The transplants were recovered 8 weeks post transplantation, fixed and decalcified in 0.25 M EDTA (Sigma-Aldrich, St. Luis, MO), cut into halves, and embedded into a single paraffin block so that the largest surface areas were sectioned. Three sections separated by 100-µm steps were prepared from each block and stained with hematoxylin and eosin. Two independent investigators masked to the groups of cells estimated the degree of bone formation using a semi-quantitative scale as previously described [23]. The average bone formation score was calculated for each transplant and for each experimental group. The extent of hematopoiesis (a measure of the presence of the stem cell within the BMSC population) in the ossicle was also evaluated and scored by microscopy.

### CD146 determination

For FACS, the entire cell suspension was pelleted, resuspended and preincubated in PBS/1% BSA for 30 min on ice with regular mixing (blocking). After washing in PBS, cells were centrifuged, and the pellet was resuspended in PBS and centrifuged again. Subsequently, they were incubated with PE-conjugated anti-CD146 (clone P1H12 monoclonal antibody BD Biosciences, 20 µl per 1×10^6^ nucleated cells) for 30 minutes on ice, washed twice with PBS/1% BSA, and resuspended in the desired volume of the same buffer. After washing, CD146^+^ and CD146^−^ fractions were separated using a FACS DIVAntageSE flow cytometer (BD Biosciences Labware, San Diego, CA) or alternatively, cell suspensions were used for flow cytometry analysis. Expression of markers was assessed by using a FACS Calibur flow cytometer and CellQuest software (Becton Dickinson Biosciences, San Diego, CA).

### GFP staining

Recovered transplants were fixed, decalcified in 0.25 M EDTA (Sigma-Aldrich, St. Luis, MO), cut into halves, embedded in paraffin blocks and sectioned. Deparaffinization and rehydration of the 10-µm paraffin sections was followed by inhibition of endogenous peroxidase and antigen retrival. Sections were incubated for 2 hours with 10% bovine serum albumin in PBS and then incubated overnight with the primary anti-GFP antibody (0.5 µg/ml, Millipore, Billerica, MA). The sections were incubated in the biotinylated secondary antibody (anti-rat-IgG peroxidase conjugate, Sigma, St. Louis, MO, 1∶200), treated with ABC reagent (Elite Vector Kit, Vector Laboratories, Burlingame, CA) and the tissue-bound peroxidase was developed was developed using diaminobenzidine.

### Gene microarrays and statistical analyses

Total RNA from BMSCs of three donors (FePro labeled, gold nanoparticle labeled and unlabeled control) was extracted using Trizol reagent and amplified into anti-sense RNA (aRNA) as previously described [24]. For comparison, total RNA extracted from human embryonic stem cells (hES) WA (H9) and adult cells: fibroblasts, endothelial cells and smooth muscle cells was amplified using the same procedure. Total RNA from peripheral blood mononuclear cells (PBMCs) pooled from six normal donors was amplified into aRNA to serve as the reference. Both reference and test aRNA (6 µg of each sample) were directly labeled using ULS aRNA Fluorescent Labeling kit (Kreatech, Salt Lake City, UT) and co-hybridized to a custom-made 36K oligo-based microarray platform encompassing the whole human genome. The arrays were printed in the Infectious Disease and Immunogenetics Section ofTransfusion Medicine, Clinical Center, NIH (Bethesda, MD) using a commercial probe set which contains 35,035 oligonucleotide probes, representing approximately 25,100 unique genes and 39,600 transcripts (Operon Human Genome Array-Ready Oligo Set version 4.0, Huntsville, AL). Hybridization was carried out at 42°C for 18 to 24 hours and the arrays were then washed and scanned on a Gene-Pix scanner Pro 4.0 (Molecular Devices, Downingtown, PA).

The resulting jpeg and gene expression data files were deposited in a microarray database (mAdb) (http://nciarray.nci.nih.gov) and further analyzed using BRBArrayTools developed by the Biometric Research Branch, National Cancer Institute (http://linus.nci.nih.gov/BRB-ArrayTools.html). Briefly, the raw data set was filtered according to standard procedure to exclude spots with minimum intensity and size. The filtered data were normalized using Lowess Smoother. Differentially expressed genes were identified using F-tests with a P-value cutoff of 0.01; P-values were adjusted for multiple comparisons by False Discovery Rate <0.05. Clustering and visualization of expression profiles was preformed with Cluster and Treeview software (http://rana.lbl.gov/EisenSoftware.htm) [25]. All the data is MIAME compliant. The entire microarray dataset is available at http://www.ncbi.nlm.nih.gov/geo/(GSE20431).
